# Screening for depression at the primary care level: Evidence for policy decision-making from a facility in Pretoria, South Africa

**DOI:** 10.4102/safp.v63i1.5217

**Published:** 2021-01-27

**Authors:** Bahupileng L. Mashaba, Saiendhra V. Moodley, Neo R.T. Ledibane

**Affiliations:** 1 School of Health Systems and Public Health, Faculty of Health Sciences, University of Pretoria, Pretoria, South Africa; 2 Gauteng Department of Health, Pretoria, South Africa

**Keywords:** depression, screening, mental health, PHQ-9, primary care, primary healthcare

## Abstract

**Background:**

Depression is a serious public health issue that has clinical, social and economic implications. Adult patients attending a primary healthcare (PHC) facility were screened in order to estimate the prevalence of depressive features and identify potential risk factors for screening positive.

**Methods:**

This was an analytical cross-sectional study conducted at a clinic in Pretoria, South Africa. A self-administered questionnaire, which included the Patient Health Questionnaire-9 (PHQ-9) screening tool, was completed by patients attending the clinic. A PHQ-9 score of less than five was deemed as a negative screen for depressive features; with a score of five or more being considered a positive screen. A multivariate logistic regression model was developed to identify factors associated with a positive screen for depressive features.

**Results:**

A total of 199 patients participated and the proportion screened positive for depressive features using the PHQ-9 tool was 46.23% (*n* = 92). Employed participants had significantly lower odds (odds ratio [OR] = 0.48; 95% confidence interval [CI]: 0.25 – 0.94) of screening positive, whilst the participants with significantly higher odds were those with co-morbidities (OR = 2.12; 95% CI: 1.08 – 4.17) and a history of stressful life events (OR = 3.21; 95% CI: 1.64 – 6.28).

**Conclusion:**

Depression appears to be a significant problem in PHC settings in South Africa. Screening for depressive features at primary level, targeting those with chronic medical conditions, history of recent stressful life events and other known risk factors may improve detection rates, lead to earlier diagnosis and improved health outcomes.

## Introduction

South Africa is experiencing a health transition with a quadruple burden of communicable, non-communicable, perinatal and maternal, and injury-related disorders.^1,2^ Mental disorders contribute to the non-communicable disease burden. Target 4 of Goal 3 of the Sustainable Developmental Goals (SDGs), focuses on mental health and it states ‘by 2030 reduce by one-third premature mortality from NCDs through prevention and treatment, and promote mental health and wellbeing’.^3^

Major depressive disorder (MDD) is a mental disorder characterised by changes in affect, mood, neurovegetative function, cognition and psychomotor activity.^4^ The assessment is based on the Diagnostic and Statistical Manual of Mental Disorders (DSM-V) criteria to diagnose MDD.^5^ The presence of five or more out of nine symptoms (depressed mood, loss of interest/pleasure; insomnia/hypersomnia, psychomotor retardation/agitation, change in weight/appetite, loss of energy/fatigue, worthlessness/guilt) which must include depressed mood or loss of interest/depressed mood in the same 2-week period is diagnostic^5^ of depressive disorder. The lifetime prevalence of major depression in South Africa has been reported at 9.7% in 2009 and the 12-months prevalence at 4.9% which is comparable with other countries.^6^ Acccording to South African Stress and Health (SASH), the prevalence of MDD in SA was 9.8% across all ages with highest prevalence of 14.6% in Free State,^7^ whereas Cholera et al,^8^ showed a prevalence of 11.8% amongst the human immunodeficiency virus (HIV) population group using the Mini International Neuropsychiatric Interview (MINI)-defined current major depressive episode (MDE).

Depression poses serious public health challenges globally.^9^ It affects the social, economic and clinical aspects of individuals resulting in impaired physical health, poor health behaviours, increased financial costs, and diminished role functioning.^9,10,11,12,13,14,15^ Depression affects all aspects of life including education, marriage, parenting skills and employment.^7^ Depression is associated with poor health-related quality of life (HRQOL) and it increases the number of years of life lived with disabilities (YLDs).^11^ The years lived with disability (YLDs) for MDD in South Africa, has increased by 58% from 408 in 1990 to 578 YLDs in 2013.^12^ According to the South African Drug and Anxiety Group (SADAG), depression cost the country about R218 billion because of presentism (attending work whilst unwell: R190b) and absenteeism (unscheduled absence from work: R28b).^13^

The components of the social determinants of mental health include the following: demographic factors, economic factors, neighbourhood, environmental events and sociocultural factors.^14^ An individual’s mental health is moulded by social, economic and physical environments that interrelate variably at different life stages.^15^ The depressive symptoms are affected independently by both negative and positive life events.^16^ Findings by Mungai et al.^17^ showed that depression is associated with socio-economic status, especially in individuals with lower education levels, low income, living in poverty and people affected by crime and violence. Stressful life events such as family deaths, break-up of the family unit, hospitalisation, incurable diseases, as well as environmental stress such as violence, crime, migration and urbanisation,^18,19^ have been shown to precede episodes of depression.^19^ Family and personal history of depression or substance abuse predisposes one to depression.^20^

Primary healthcare (PHC) is the initial area of contact between patient and the health care system,^21,22^ and it could play a pivotal role in the management of depression. Depression can be detected and treated at a PHC setting where treatment is feasible, affordable and effective.^23^ Primary healthcare is the relevant area for screening, diagnosing and treating depression, but because of increasing workload at PHC settings, limited human resources and lack of screening tools, little time is available to screen for mental disorders and depression may go undiagnosed.^24^ Patients with depression are frequent users of medical services, therefore, PHC clinicians should actively seek to detect depressive disorders to prevent suicide and reduce healthcare costs.^24^

South Africa has made PHC universally accessible to individuals and families in the communities as per the World Health Organization (WHO) recommendation.^1^ The South African government and the Department of Health (DOH) specifically have adopted different strategies to improve mental health: the Mental Health Care Act, 2002 (MHCA), the new Mental Health Policy Framework (MHPF) and Strategic Plan 2013–2020, and the WHO mental health Gap Action Program (mhGAP). According to the MHCA, mental health is a public health priority including human rights for those who need mental healthcare and rights to access to care.^1^ The MHPF aims at task-sharing and integration of mental health into PHC services.^1^ The mhGAP aims to integrate the mental health services into PHC with delivery through the district health services (DHS) and the PHC support team.^3^ To enhance good mental health at the PHC level, the South African health system has linked PHC with specialists, training of healthcare workers and introduction of Primary Care-101 (PC-101) and the standard treatment guidelines and essential medicines list (EML) book.^3^ The PC-101 assists with identification and management of chronic diseases to strengthen and support clinical decisions.^25^

Studies by Beard et al.,^26^ Cholera et al.,^8^ Grobler.^7^ and Siu et al.^27^ highlighted the importance of using primary care in identifying patients with depressive symptoms and they have identified the use of the Patient Health Questionnaire-9 (PHQ-9) as a reasonable screening tool in accurately classifying cases of depression and can be easily implemented by lay health workers. Findings by Cholera et al.^8^ and Ganguly et al.^28^ showed that PHQ-9 has been used in resource-limited settings such as sub-Sahara Africa. South Africa has implemented different strategies to improve mental health care within the country but there has not been a focus on routine screening at PHC facilities. A few studies have been done in South Africa focusing on validity of PHQ-9 as a screening tool for depression, but no recommendations were made for routine screening.^8,29^ A study by Anderson et al.^30^ in the Eastern Cape revealed that adult patients do not self-report depressive features despite going to PHC facilities frequently.

This study screened for depressive features amongst adult patients who attended a PHC facility in Pretoria, Tshwane, South Africa from 01 February 2018 to 28 February 2018. We determined the proportion of patients who screened positive for depressive features, and the risk factors associated with a positive screen for depressive features amongst patients. We envisage that the findings of our study may provide evidence to assist in decision-making with respect to screening for depression at PHC level.

## Methods

### Study design and setting

The study used an analytical cross-sectional study design. It was conducted at a PHC clinic located in Tshwane District in northern Gauteng Province in Pretoria, South Africa. Medical services rendered at the facility include maternal and child health, communicable diseases, non-communicable diseases and general PHC services.

### Study population and sampling

The study population comprised patients 18 years and older, who sought medical services at the clinic. Stable, down-referrals from the psychiatric hospital, patients already diagnosed with depression at the clinic and patients younger than 18 years of age, were excluded from the study. A sample size of 200 was calculated from the total head count of 7899, from April 2015 to April 2016. A systematic random sampling technique was employed, where every 5th patient was selected with a random starting point between one and 10.

### Measurements and data collection

A modified PHQ-9 questionnaire was used as the screening tool.^8,23^ It was based on the two main questions from the DSM-IV criteria for major depression.^23,25^ The PHQ-9 screening tool has proven to be valid and reliable for use in primary care,^23,31^ and can be used in high income^20,31^ and resource-limited settings^8,29^ as well. It is short, simple and easy to administer.^8,29^ The first part of our questionnaire focused on the demographic data, potential risk factors and medical history. The second part of the questionnaire focused on depressive features for at least 2 weeks (PHQ-9), as well as functional health assessment.

The questionnaires, after completion were given to the healthcare professional attending to the patient who scored them and placed them in the questionnaire boxes supplied for each consulting room. The questionnaire was self-administered, but participants who did not understand English were assisted by either the trained assistant or the principal investigator individually in a private room to complete the questionnaire prior to their consultation with the healthcare professional. An interpreter was sought where needed, because of the multi-lingual nature of the patients seen at the facility. The questionnaire took approximately 20–40 min to complete.

### Data analysis

The raw data were entered into EpiData version 3.1, using a double-entry process and then exported to Microsoft Excel for cleaning and coding. All the analyses were performed using Stata version 14 (StataCorp., College Station, TX, United States [US]). Proportions were calculated for screening outcome which was considered as either positive for depressive features (score ≥ 5), or negative for depressive features (score < 5) (Table 2). Logistic regression models (univariate and multivariate) were used to determine the factors associated with depressive features and a value *p* < 0.25 was used for including variables from the univariate analysis in the multivariate model.

### Ethical consideration

The ethical principles for medical research involving human subjects were applied during this study according to the Declaration of Helsinki. Approval to conduct the research study at the facility was granted by the Tshwane District Research Committee and registered on the National Health Research Database (NHRD) (reference number 100/2017). The University of Pretoria Faculty of Health Sciences Research Ethics Committee approved the study protocol. The questionnaires used were anonymous to ensure confidentiality and informed consent was sought and obtained from the patients.

## Results

Two hundred (200) patients were invited to participate in the study and of those, 199 participants completed the questionnaire with the response rate of 99.5%. The participants enrolled were between the ages of 18 and 79 years with a mean of 40.17 years. The female participants (79.90%) outnumbered their male counterparts. The proportion of participants that were married and single was similar at 47.00% and 44.95%, respectively. The majority of participants identified their race as African (90.26%). Less than half of the participants (47.37%) were employed and 71.63% of the participants had an income less than R3500 (national minimum wage). Most of the participants resided in urban areas (75.92%) with the highest proportion (32.16%) of participants identifying Gauteng Province as their province of origin. Almost a quarter of participants (24.62%) indicated that they were not from South Africa originally. Table 1 depicts the socio-demographic characteristics of the participants.

**TABLE 1:** Socio-demographic characteristics of participants.

Characteristics	*n*	%
**Gender (*N* = 199)**		
Female	159	79.90
Male	40	20.10
**Marital status (*N* = 198)**		
Married	93	47.00
Single	89	44.95
Divorced	11	5.56
Separated/widowed	5	2.53
**Race (*N* = 195)**		
African	176	90.26
Coloured	6	3.07
Indian	2	1.03
White	11	5.64
**Highest education (*N* = 197)**		
No schooling	4	2.03
Primary	19	9.64
Secondary	130	65.99
Tertiary	44	22.34
**Employment (*N* = 190)**		
Yes	90	47.37
No	100	52.63
**Current occupation (*N* = 82)**		
Contractual workers	45	54.88
Administrators	14	17.07
Other occupation	23	28.05
**Income per month (*N* = 141)**		
0–1000	38	26.95
1001–3500	63	44.68
3501–10 000	28	19.88
> 10 000	12	8.51
**Area of residence (*N* = 191)**		
Urban	145	75.92
Rural	27	14.14
Peri-urban	19	9.95
**Province of origin (*N* = 199)**		
Gauteng	64	32.16
Limpopo	30	15.08
Mpumalanga	24	12.06
Other SA provinces	32	16.08
Non-SA	49	24.62

SA, South Africa.

The PHQ-9 categories (based on the sum of the number of depressive features present) and the percentages of participants in each category are shown in Table 2. The majority of participants (53.77%) had a PHQ-9 score of 0–4 whilst 2.51% of participants fell in the ‘severe’ PHQ-9 category of 20–27. The PHQ-9 scores were then categorised into either a negative screening outcome (score of 0–4) or positive screening outcome (score of 5–27). The proportion of participants that screened positive using the PHQ-9 screening tool was 46.23% (*n* = 92).

**TABLE 2:** Screening outcomes for depressive features using patient health questionnaire-9 score (*N* = 199).

PHQ-9 score	PHQ-9 categories	Screening outcome	*N*	%
0–4	None-minimal	Negative	107	53.77
5–9	Mild	Positive	52	26.13
10–14	Moderate	Positive	22	11.06
15–19	Moderately severe	Positive	13	6.53
20–27	Severe	Positive	5	2.51

PHQ, patient health questionnaire.

Based on the univariate logistic regression (Table 3), the variables found to be significantly associated with a positive screen for depressive features were a history of stressful life events (*p* = 0.001), drink (alcohol) index (*p* = 0.017), co-morbid disease (*p* = 0.052) and country of origin (*p* = 0.006). In addition to these, marital status, race, employment and history of trauma were retained for multivariate modelling purposes using a *p*-value of 0.25

**TABLE 3:** Univariate logistic regression results for factors associated with screening positive on the patient health questionnaire-9.

Variable	OR	*p*-value	95% CI
**Age (years)**			
18–35	1.00	-	-
36–55	1.00	0.993	0.53–1.90
56 and above	0.80	0.573	0.38–1.71
**Gender**			
Female	1.00	-	-
Male	0.83	0.59	0.41–1.67
**Marital status**			
Single	1.00	-	-
Married	0.84	0.560	0.47–1.51
Divorced/separated	0.33	0.068	0.10–1.09
**Race**			
African	1.00	-	-
White/coloured/Indian/other	0.50	0.150	0.19–1.28
**Highest education level**			
Degree/diploma	1.00	-	-
Grade 4–10	1.25	0.527	0.63–2.46
No schooling	4.33	0.220	0.42–45.06
**Employed**			
No	1.00	-	-
Yes	0.65	0.138	0.37–1.15
**Income per month**			
R0.0 – R3500.00	1.00	-	-
R3501.00 – above	0.69	0.331	0.33–1.46
**Area of residence**			
Peri-urban	1.00	-	-
Rural	1.20	0.765	0.37–3.88
Urban	0.93	0.879	0.36–2.42
**Family history of depression**			
No	1.00	-	-
Yes	1.39	0.359	0.69–2.83
**Co-morbid disease**			
No	1.00	-	-
Yes	1.75	0.052	0.99–3.07
**History of stressful life events**			
No	1.00	-	-
Yes	3.80	0.001	2.09–6.94
**Drink index**			
No	1.00	-	-
Yes	2.25	0.017	1.16–4.37
**Used drugs**			
No	1.00	-	-
Yes	1.37	0.58	0.44–4.24
**Country of origin**			
South Africa	1.00	-	-
Non-SA	0.39	0.006	0.19–0.78

OR, odds ratio; SA, South Africa; CI, confidence interval.

The multivariate logistic regression model is displayed in Table 4. Employed participants had significantly lower odds (OR = 0.48) of screening positive (*p* = 0.033) for depressive features whilst the participants with significantly higher odds of being screened positive were those with co-morbidities (OR = 2.12; *p* = 0.029) and a history of stressful life events (OR = 3.21; *p* = 0.001). The post regression test, with the area under the receiver operating characteristic (ROC) curve of 0.750, reflected that the model was acceptable as 75% of the observations were correctly represented, with the Pearson’s chi-square of 0.022.

**TABLE 4:** Multivariate logistic regression model for factors associated with screening positive on the patient health questionnaire-9.

Variable	OR	*p*-value	95% CI
**Marital status**			
Single	1.00	-	-
Married	1.47	0.285	0.73–2.96
Divorced/separated	0.39	0.167	0.10–1.49
**Employed**			
No	1.00	-	-
Yes	0.48	0.033	0.25–0.94
**Highest education level**			
Degree/diploma	1.00	-	-
Grade 4–10	0.87	0.729	0.39–1.96
No schooling	1.95	0.618	0.14–27.02
**Co-morbid disease**			
No	1.00	-	-
Yes	2.12	0.029	1.08–4.17
**History of stressful life events**			
No	1.00	-	-
Yes	3.21	0.001	1.64–6.28
**Drink index**			
No	1.00	-	-
Yes	1.86	0.106	0.88–3.93
**Country of origin**			
South Africa	1.00	-	-
Non-SA	0.51	0.106	0.23–1.15
**Race**			
African	1.00	-	-
White/coloured/Indian/other	0.57	0.302	0.19–1.67

OR, odds ratio; SA, South Africa; CI, confidence interval.

## Discussion

The proportion of participants screened positive for depressive features in our clinic-based study population was 46.23% using a PHQ-9 score of five or more. The prevalence of depression in a study amongst adults (18–40 years) in the Eastern Cape Province was discovered to be 31.4%, where the mini international neuropsychiatric questionnaire was used.^30^ A study by Gray et al.^32^ found a prevalence of 35% amongst community members with both hypertension and depression. Another study in Lilongwe using a PHQ-9 score of 10 or more, reported the prevalence of depressive disorders amongst people living with HIV to be 12%.^33,34^ In comparison, 20.10% of participants in our study had a PHQ score of 10 or more.

Since the PHQ-9 is a screening test for depression and does not constitute a formal diagnosis of depression, it is possible that ‘false positives’ may have contributed to the high proportion screened positive in our study. A Tanzanian study on validation of PHQ-9, showed it to have a reasonable sensitivity (78%) and high specificity (87%) at a cut off score of nine.^34^ Using a score of five or more for a positive screen in our study would have increased the sensitivity but reduced the specificity of the PHQ-9. Besides ‘false positives’, it is a reasonable assumption that patients sampled at a clinic would have a higher prevalence of depression than the general population, as they are more likely to have known risk factors such as chronic illnesses. The high proportion screened positive in this study could also potentially be attributed to the socio-demographic composition of our participants being different from the general population. For example, the proportion of females in our study was 79.90% compared to approximately 51% in the South African population in 2018 and the proportion of unemployed persons in our sample was higher than the unemployment rate for South Africa in 2018, even after taking into account that some of the unemployed participants in our study would have been pensioners.^35^

The only socio-demographic factor in our study found to be significantly associated with a positive screen for depressive features following multivariate logistic regression was employment status. Being employed was protective with an odds ratio of 0.48 (*p* = 0.033). A Canadian study by Romans et al.^36^ supported the fact that unemployment increases the rate of depression. Whilst confirming the evidence in the literature, this finding is concerning given the high levels of unemployment in South Africa.

The presence of comorbidities have a negative impact on individual health and public health.^1^ Depression is a co-morbid disorder with chronic conditions such as arthritis, asthma, cancer, cardiovascular disease, diabetes, hypertension, chronic respiratory disorders, HIV/acquired immune deficiency syndrome (AIDS) and chronic pain.^10,13,23,25^ The participants with comorbidities had 2.12 higher odds of screening positive for depressive features than those without co-morbidities (*p* = 0.029). This may be attributable to the poor adherence to self-care, increased medical costs, functional impairment, increased medical symptoms burden and increased risk of morbidity and mortality.^37^

The odds of screening positive for depressive features were 3.21 higher when there was the presence of stressful life events compared to their absence. The common stressful life events experienced by the participants were social issues (78.13%), medical events (72.92%) and traumatic life events (69.30%). A study by Lewis et al.^19^ supported the fact that the stressful life events such as death, breakup of extended families, migration, immigration and hospitalisation can lead to depression.

Whilst the substantial proportion screened positive for depressive features in our study may not reflect the actual prevalence in the catchment population, it does suggest that depression is a significant issue in this community. Whilst an argument can be made that depression fulfils the criteria that comprise Wilson and Jungner’s principles of screening, the practicality and feasibility of routinely screening all adult patients presenting themselves at PHC facilities for depression needs to be considered.^38^

Routine screening for depression at pramary care level has previously been recommended in the United States and Canada.^39^ The United State Preventative Task Force (USPSTF) recommends screening for depression in the general population using PHQ-9.^40,41^ The implementation should be done where there are adequate systems to ensure accurate diagnosis, effective treatment and appropriate follow up.^39,40,41^ The benefits of routine screening include early detection, improved clinical outcome and improved detection of undetected cases.^40^ Thombs et al.^39^ have outlined some disadvantages of routinely screening for depression. Screening can be harmful if some patients who were incorrectly identified are treated with antidepressants and are exposed to the side effects of the drugs.^39^ If the health system is struggling financially, introducing routine screening for depression may place additional pressure on the health system.^39^ Routine screening can result in the nocebo effect amongst some patients resulting in development and worsening of symptoms.^39^

Keeping the advantages and disadvantages of routinely screening all primary care patients for depression in mind, targeted screening at primary care level aimed at specific at-risk groups may be the appropriate policy option at this juncture. Based on our study findings, we recommend the development of a routine depression screening protocol for patients with chronic medical conditions. In addition, clinicians should routinely enquire about stressful life events as part of the patient history followed by screening for depression if this is warranted, and clinicians should maintain a high index of suspicion of depression in patients with known risk factors such as unemployment. Whilst the PHQ-9 screening tool was used for our study, further research on the most appropriate tool to use in South African PHC settings is warranted. Once agreed upon, the screening tool should be accessible to clinicians at all PHC facilities in South Africa with appropriate diagnosis, treatment and referral protocols in place for patients screened positive.

### Limitations

This was a cross-sectional study and it is, therefore difficult to assess temporality (e.g. employment status and positive PHQ-9 screen) and causal relationships. There is the possibility that there may have been under-reporting on some sensitive variables because of social desirability, for example, substance use; thus constituting information bias. The questionnaire was available in English only although the effect of inaccuracies because of verbal translation to the overall results would have been minimal as only two participants did not understand English. The study was conducted at a single PHC facility in Tshwane. The proportion of patients screened positive is likely to be as a result of the patient profile at this facility and should be generalised with caution. With respect to the risk factors identified, this is supported by the literature and these findings are more likely to be generalisable.

## Conclusion

A large proportion of participants in our study screened positive on the PHQ-9. This was associated with employment status, co-morbidities and recent stressful life events. Screening for depressive features at PHC facilities targeting those with chronic medical conditions, history of recent stressful life events and other known risk factors may improve detection rates, lead to earlier diagnosis and improved patient outcomes.
